# Low serum levels of vitamin D significantly increase the risk of death in older adults with hip fractures: a prospective cohort

**DOI:** 10.1590/0100-6991e-20223054

**Published:** 2022-03-19

**Authors:** MARCELO TEODORO EZEQUIEL GUERRA, MARIO WAGNER, ALFONSO VARGAS, CARLOS ROBERTO GALIA

**Affiliations:** 1 - Universidade Luterana do Brasil - Canoas - RS - Brasil; 2 - Universidade Federal do Rio Grande do Sul - Porto Alegre - RS - Brasil

**Keywords:** Albumins, Aged, Mortality, Nutritional Status, Vitamin D, Albumina, Envelhecimento Mortalidade, Estado Nutricional, Vitamina D

## Abstract

**Objective:**

to evaluate the relationship between 25(OH)D_3_ levels and fatal outcome in patients over 60 years of age undergoing surgical repair of hip fractures.

**Methods:**

prospective cohort of patients undergoing surgical repair of hip fractures. At admission, 25(OH)D_3_ levels were measured, among other parameters. Patients were followed for at least 1 year, and incident mortality was recorded.

**Results:**

209 patients were included in the study, with a mean age of 79.5 ± 7.6 years among survivors and 80.7 ± 8.2 years among those who died in the first postoperative year (p=0.346). The 25(OH)D_3_ levels of survivors were significantly higher than those of patients who died (p=0.003). After adjusting for confounding variables, 25(OH)D_3_ levels below 12.5ng/mL were significant risk factors regardless of mortality (adjusted OR: 7.6; 95% CI: 2.35 to 24.56).

**Conclusions:**

our data show that serum 25(OH)D_3_ levels below 12.5ng/mL significantly and independently increased the risk of mortality in the first year after surgical repair of low-energy hip fracture in patients over 60 years of age in the geographic region where this study was conducted. Low albumin also showed a significant association with mortality in these patients. All other factors had no significant associations.

## RESUMO

**Objetivo:**

avaliar a relação entre os níveis de 25(OH)D_3_ e o desfecho fatal em pacientes acima de 60 anos submetidos a correção cirúrgica de fratura de quadril.

**Métodos:**

coorte prospectiva de pacientes submetidos a correção cirúrgica de fraturas de quadril. À admissão, foram medidos os níveis de 25(OH)D_3_, entre outros parâmetros. Os pacientes foram acompanhados por, pelo menos, um ano e a mortalidade foi registrada.

**Resultados:**

foram incluídos 209 pacientes no estudo, com média de idade de 79,5 ± 7,6 anos entre os sobreviventes e 80,7 ± 8,2 anos entre os que morreram no primeiro ano de pós-operatório (p=0,346). Os níveis de 25(OH)D_3_ dos sobreviventes foram significativamente maiores do que os dos pacientes que morreram (p=0,003). Após o ajuste para variáveis de confusão, níveis de 25(OH)D_3_ abaixo de 12,5ng/mL foram fator de risco significativo, independentemente da mortalidade (OR ajustado 7,6; IC 95% 2,35 24,56).

**Conclusões:**

níveis séricos de 25(OH)D_3_ abaixo de 12,5ng/mL aumentaram significativa e independentemente o risco de morte no primeiro ano após o reparo cirúrgico de fratura de quadril de baixa energia em pacientes com mais de 60 anos de idade na região geográfica onde este estudo foi realizado. A albumina baixa também teve associação significativa com a mortalidade nesses pacientes. Todos os outros fatores não tiveram associações significativas.

## INTRODUCTION

Hip fractures have a significant impact on health both at the society^1^ and individual level^2^ ^,^ ^3^. Outcomes vary widely from country to country^4^, from region to region, and across ethnicities^5^. In southern Brazil, hip fractures are highly prevalent and carry high mortality rates in the white population^6^. Other factors are also important, such as the influence of climate, with a higher incidence of falls and fractures in the winter months^7^ ^,^ ^8^.

The morbidity and mortality rates of this condition vary according to several risk factors^9^ ^,^ ^10^. Among these, those related to nutritional status interfere with both clinical outcomes and mortality rates^11^ ^,^ ^12^. Low albumin levels are associated with unfavorable outcomes^13^, whereas low 25(OH)D_3_ levels are associated with an increased overall risk of fractures in older persons^14^ ^-^ ^17^. Some studies have analyzed possible associations of 25(OH)D_3_ with morbidity and mortality after surgical treatment of hip fractures^18^ ^,^ ^19^.

In caring for older patients with hip fracture, knowing the factors that can identify those at increased risk of death provides a better understanding of the clinical conditions that of these patients, which can improve the care of this complex patient population. Within this context, the present study was designed to assess the association between preoperative serum 25(OH)D_3_ levels and 1-year mortality in older adults (age >60 years) undergoing surgical repair of hip fractures in the southern region of Brazil.

## METHODS

### Study design

Prospective cohort.

### Participants

This study builds upon a previous retrospective cohort study^17^, which after the publication of these results, other patients were included in the cohort. The inclusion criteria were patients aged >60 years who underwent surgical treatment for low-energy hip fractures between January 2015 and December 2016. Patients with refracture, high-energy trauma, or concomitant infectious diseases, patients who did not have 25(OH)D_3_ levels measured preoperatively, and those who were nonambulatory at baseline were excluded. 

**Figure 1:**
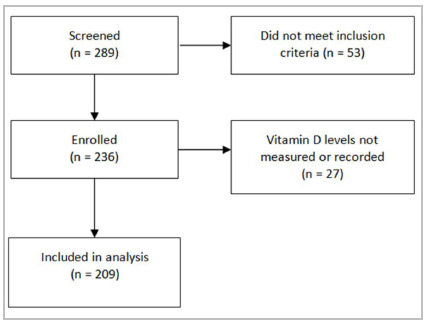
Flow diagram of participant inclusion.

After application of the inclusion and exclusion criteria, 236 additional patients were enrolled. Of these, 27 patients did not have 25(OH)D_3_ levels recorded. Therefore, 209 patients were analyzed in the present study (Figure 1).

### Ethics

The study protocol was approved by the institutional Human Subject Research Ethics Committee (IRB equivalent) on June 26, 2017. The present study was conducted in accordance with the Brazilian National Health Council Resolution No. 466/12. All procedures were in accordance with the ethical standards of the institutional research committee and with the 1964 Helsinki declaration and its later amendments or comparable ethical standards. Written informed consent was obtained from all participants or their caregivers prior to enrollment. Patients or caregivers were contacted by telephone. If telephone contact was unsuccessful, an active search was conducted for mortality information in official vital records.

### Procedures

**Figure 2:**
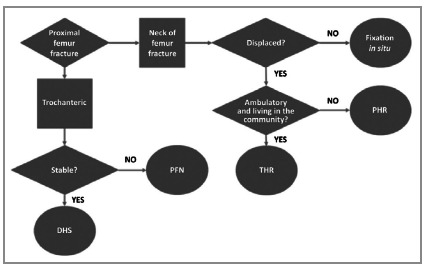
Institutional protocol for management of hip fractures in older adults. DHS, dynamic hip screw; PFN, proximal femoral nail; THR, total hip replacement; PHR, partial hip replacement.

All patients were treated following a protocol for the care of older adults with hip fractures. On the day of admission, alongside a routine preoperative workup, serum levels of 25(OH)D_3_ and other parameters were measured. The surgical procedure indicated per protocol (Figure 2) was performed by an experienced orthopedic surgeon. 

Since the preoperative period, all patients were followed by a team of hospitalists. Upon admission, hospital social workers assessed each patient’s conditions to ensure a safe return to their place of origin (home or otherwise). Postoperatively, patients were followed by the hospital Orthopedics and Trauma Surgery team for at least one year. 

### Outcome Variables

The primary outcome of interest was mortality.

### Assessment of Covariates

As noted above, all patients had serum levels of several markers measured upon admission. Information was collected on the following variables: smoking and alcohol intake; preexisting diagnosis of dementia, hypertension, diabetes mellitus, chronic kidney disease, AIDS, hepatitis, delirium, and deep vein thrombosis; weight and height; C-reactive protein, vitamin D, albumin, urea, creatinine, and glucose; and complete blood count with differential. As per institutional protocol, a cardiologist calculated the score for risk of cardiac complications proposed by Lee et al^20^.

### Sample Size

Assuming a 1-year mortality rate of 20% for the reference group (serum 25(OH)D3 >20 ng/mL) and 40% for the exposed group (serum 25(OH)D_3_ ≤15ng/mL), 82 patients per group would provide a statistical power of 80% with a two-sided type I error of 0.05. To allow for multivariable statistical modeling, sample size was increased by 20%. Therefore, our study was designed to include a total of 200 patients (100 patients per group).

After analyzing several intervals of serum 25(OH)D_3_ levels, we chose the cut-offs of <12.5ng/mL, 12.5-25ng/mL, and >25ng/mL because they resulted in intervals of greater statistical significance.

## RESULTS

Of 289 patients, 236 met the inclusion criteria. Of these, 209 had serum 25(OH)D_3_ levels measured and were included in the present analysis (Figure 1). Among the fractures, 44% were neck of femur fractures, 47% were trochanteric, and 9% were subtrochanteric. Fifty-two patients died during the first postoperative year, which represents a 1-year mortality rate of 22%. Of these, 44% had neck of femur fractures, 50% had trochanteric fractures, and 3% had subtrochanteric fractures. A body mass index (BMI) <18.5kg/m^2^ was observed in 9% of patients. Overall, 61% had serum 25(OH)D_3_ levels below 20ng/mL. Serum 25(OH)D_3_ levels were significantly higher in survivors than in those who died within 1 year of surgery (p=0.003). Likewise, albumin levels were significantly lower in patients who died in the first postoperative year than in survivors (p=0.03). There was no significant difference in fracture region, BMI, neutrophil-to-lymphocyte ratio, C-reactive protein, or blood glucose levels between patients who died and those who survived (Table 1).

**Table 1:** Baseline characteristics of patients.

Characteristics	Alive at 1 year	Dead at 1 year	
	n=184	n=52	p
Mean age, yrs (SD)	79.5±7.6	80.7±8.2	0.346
Female sex, n (%)	139 (76.0)	37 (71.2)	0.474
Fracture region, %			0.693
Neck of femur	81 (44.3)	23 (44.2)	
Intertrochanteric	85 (45.9)	26 (50.0)	
Subtrochanteric	18 (9.8)	3 (5.8)	
BMIa, kg/m^2^, n (%)	n=153	n=47	0.525
<18.5	17 (11.1)	2 (4.3)	
18.5 to <25.0	87 (56.9)	27 (57.4)	
25.0 to <30.0	39 (25.5)	14 (29.8)	
≥30	10 (6.5)	4 (8.5)	
25-(OH)D3 level, ng/mL	n=162	n=47	0.003
<10	8 (4.9)	11 (23.4)	
10 to <20	85 (52.5)	23 (48.9)	
20 to <30	47 (29.0)	10 (21.3)	
>30	22 (13.6)	3 (6.4)	
	n=180	n=52	
Albumin, g/dL	3.47±0.63	3.24±0.78	0.03
	n=178	n=52	
NLRb, mg/L	5.14 (3.23; 7.28)	5.55 (3.05; 7.98)	0.703
	n=172	n=44	
CRPc	20.8 (4.0; 55.8)	13.7 (4.7; 54.4)	0.694
	n=178	n=51	
BGd, mmol/L	132±42	138±74	0.515

^*a*^ *Body mass index.*

^*b*^ *Neutrophil-to-lymphocyte ratio.*

^*c*^ *C-reactive protein.*

^d^Blood glucose level.

**Figure 3:**
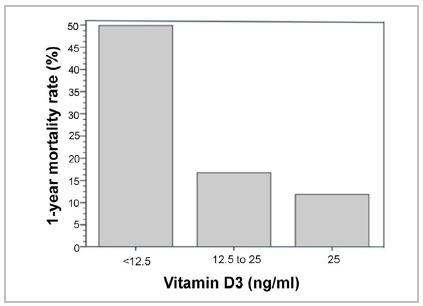
Bar plot of 1-year mortality rate and vitamin D3 level.

Regarding 25(OH)D_3_, 50% of patients with levels below 12.5ng/mL died (Figure 3), with an odds ratio (OR) of 7.40 for mortality (p=0.001). After adjusting for potential confounding effects of age, sex, and albumin level <3g/dL, OR was 7.60 (p=0.001) (Table 2).

**Table 2:** Incidence of 1-year mortality among patients undergoing hip fracture surgery, stratified by preoperative serum vitamin D levels, Porto Alegre, Brazil.

Serum vitamin D3	n	events	%	OR	95%CI	p	OR*	95%CI	p
0 to <12.5	42	21	0	7.4	2.43 - 22.51	<0.001	7.6	2.35 - 24.56	0.001
12.5 to 25	125	21	6.8	1.49	0.53 - 4.25	0.451	1.46	0.50 - 4.29	0.492
>25	42	5	1.9	1		-	1		-
Total	209	47	2.5	-	-	-			-

OR obtained in a logistic regression model adjusted for the confounding effects of age, gender, and serum albumin levels ≤ 3 g/dL.

## DISCUSSION

Hip fracture is an important public health problem worldwide. In the past five years, more than 4.846 articles with a MeSH Major Topic of Hip Fracture have been indexed in MEDLINE. However, a search query combining MeSH Major Topic Hip Fracture AND Title Risk Factors yields just over 137 articles. With a search query (“hip fractures”[MeSH Major Topic]) AND (“Vitamin D”[MeSH Major Topic], only one result was found in the past five years^21^.

To date, to our knowledge, no study has correlated serum 25(OH)D_3_ levels with mortality rate after hip fracture in South America. Considering that our state is geographically located close to the 30^th^ South parallel and the ethnic makeup of the southern Brazilian population is overwhelmingly white (>80%)^22^, studies assessing the effects of serum 25(OH)D_3_ levels in patients with hip fractures are necessary. Our sample is similar to those previously reported in the literature in terms of age and sex^18^ ^,^ ^19^.

The most striking finding of our study is the significant association between 25(OH)D_3_ levels <12.5ng/mL and risk of death in the first year after surgical treatment. We also observed a significant relationship between lower albumin levels and 1-year mortality. However, despite the close relationship between the levels of 25(OH)D_3_ and albumin, the strong association of 25(OH)D_3_ levels with mortality outcomes persisted even after adjusting the results for albumin, age, and sex.

The relationship between low serum 25(OH)D_3_ levels and all-cause mortality has been well studied^23^ ^,^ ^24^, with levels below 30ng/mL being associated with an increased risk of mortality. Other authors have also found associations between 25(OH)D_3_ levels and mortality. Toldy et al.^19^, in a case-control study, had results similar to ours. Fisher et al.^21^, in multivariate analyses, found seven variables that independently increased in-hospital mortality among patients treated for hip fracture, including serum 25(OH)D_3_ levels below 25nmol/L. Whiting et al.^18^, using data from a cohort of 23,178 individuals divided into a group without vitamin D supplementation and a group with a minimum of 800IU/day of vitamin D supplementation, showed that in men, who are known to be at higher risk of mortality due to hip fractures, the decrease in mortality risk with vitamin D supplementation was not significant. In addition to the selection bias reported in the study, supplementation was neither regular nor related to serum 25(OH)D_3_ levels. Lee et al.^25^ showed a relationship between low 25(OH)D_3_ levels and mortality, in univariate analyses. However, unlike our study, they did not find a significant relationship in the multivariate analyses.

Our results raise the issue of vitamin D replacement. A PubMed search combining (“hip fractures”[MeSH Major Topic]) AND (“vitamin D/administration and dosage”[MeSH Major Topic]) returned 3 articles^26^ ^-^ ^28^. Or et al.^26^, in addition to other measures, implemented a protocol for administration of 2000IU of vitamin D/day in patients with 25(OH)D_3_ levels above 20ng/mL. If 25(OH)D_3_ levels were below 20ng/mL, patients received a loading dose of 75,000IU. These patients were monitored by health professionals with the purpose of maintaining adherence to the protocol. Despite a decrease in mortality rates, the authors recognized the methodological weaknesses of the study. Thorpe et al.^27^ reported a decrease in the risk of hip fracture among white female vegans with vitamin D supplementation. However, they did not investigate the postoperative period and the demographic and dietary profile of the sample is very specific. Al-Khalidi et al.^28^ concluded that vitamin D supplementation is effective in the prevention of hip fractures.

Our study has limitations. The socioeconomic specificities of our region and the ethnic characteristics of our population may have caused some selection bias. However, we believe that this limitation also represents an opportunity to learn about the behavior of these factors in other scenarios.

We understand that the influence of vitamin D on the outcomes of patients with hip fractures needs to be further investigated. Some questions remain unanswered: is there room for vitamin D replacement in these patients? For all patients? At what dose? Starting when? Until when? What is the association of serum 25(OH)D_3_ levels with functional outcomes? Do we have data to implement public vitamin D replacement policies in the older population? Our data showed that low albumin levels are also associated with 1-year mortality. Although the logistic regression models did not associate the two factors, we understand that further studies are needed to assess the real role of albumin in these scenarios. Finally, our study is sufficiently powered to allow us to state that serum albumin levels below 3g/dL as well as serum 25(OH)D_3_ levels below 12.5ng/mL significantly and independently increase the risk of mortality in the first year after surgical repair of low-energy hip fracture in patients over 60 years of age.
